# Diversity in Medical Education

**DOI:** 10.3205/zma001320

**Published:** 2020-03-16

**Authors:** Sabine Ludwig, Christian Gruber, Jan P. Ehlers, Sabine Ramspott

**Affiliations:** 1 Charité – Universitätsmedizin Berlin, Berlin, Germany; 2 University of Applied Sciences, Department of Applied Health Sciences, Bochum, Germany; 3 University of Veterinary Medicine, Foundation, Hannover, Germany; 4 Witten/Herdecke University, Didactics and Education Research in the Health Sector, Faculty of Health, Witten, Germany; 5 Trillium GmbH Medizinischer Fachverlag, Grafrath, Germany

## Editorial

The existing and increasing diversity in our population and thus among patients and students requires that diversity aspects are adequately taken into account in education and training in medicine and health care professions. According to the General Equal Treatment Act (AGG), diversity comprises six categories: age, gender, ethnicity, physical impairment, sexual orientation and religion. However, many other aspects also play a role when it comes to inclusion or discrimination (e.g. socioeconomic status, education, hair colour or body shape, to name just a few).

For the quality of medical care, it is important that diversity aspects are taken into account and implemented in the curricula. In addition to curricular integration, diversity aspects should also be taken into account in study programme structures and the study environment, including family-friendly timetables, barrier-free access to classrooms and courses, and diversity sensitive admissions procedures. It is therefore necessary to “fix the content, the institution and the numbers” [1].

There are several efforts to integrate diversity aspects, both in Europe and globally. Gender-sensitive curricula have been developed in medical schools in America [2] and Canada [3], as well as in Austria, Sweden, Germany, the Netherlands and other countries [4], [5], [6], [7]. This could be achieved, for example, by means of suitable quality management instruments and approaches such as a “Gender and Diversity Change Agent” [8].

There are also increased efforts to integrate cultural competencies. Dogra et al. have developed twelve tips for culturally sensitive medical education [9]. In 2014, the Lancet Commission on Culture and Health stressed the importance of integrating cultural competencies into medical education [10]. The Committee on Cultural Competencies and Global Health from the German Association for Medical Education (GMA) has also published a position paper on the integration of cultural competencies in medical education [11].

In Europe the process of integration has been supported by a position paper of the* Standing Committee of European Doctors* (CPME) highlighting the importance of integrating sex and gender aspects into medical education and its impact on the quality of health care [http://www.cpme.eu/cpme-policy-on-sex-and-gender-in-medicine/].

Furthermore, the Committee on Gender, Diversity and Career Development in Medical Education and Training of the German Association for Medical Education (GMA) supports the integration of sex/gender- and diversity-related learning objectives into the National Competence Based Catalogue of Learning Objectives for Undergraduate Medical Education (NKLM) [http://www.nklm.de]. At the International Association for Medical Education (AMEE), there is also an *International Community of Practice* on "Diversity, Equity and Inclusion in Medical Education" promoting and supporting the further integration of diversity issues [https://amee.org/home].

Aspects of diversity are also increasingly integrated into university didactics by using sex/gender- and diversity-sensitive teaching and learning materials and sex/gender- and diversity-sensitive language [12].

Despite these measures, a survey of medical faculties in Germany has shown that the integration is “still in its infancy”. “The curricular integration of sex- and gender-specific medicine into medical curricula is very heterogeneously regulated in Germany. Comprehensive concepts for a better integration are necessary”. For example, the responsibility for sex/gender-sensitive teaching mostly lies with the teachers, and there are no central control mechanisms to monitor if sex and gender aspects are actually taught and part of the assessment. Only one faculty meets the current international assessment standards in this regard [13].

A study in the Netherlands on the integration of diversity aspects into a medical curriculum has shown that, despite supportive framework conditions, the teaching material is not yet sufficiently diversity-sensitive [14]. In addition, we don’t know the status quo of the integration of diversity aspects in the curricula of other health care professions. In conclusion, research on diversity-related competencies is yet to be enhanced and expanded.

We have compiled this special edition booklet to provide an overview of approaches that have been used, measures that have proved to be successful, what has already been achieved, and to present best practice examples and recommendations for other institutions. This special edition includes original papers, projects, reviews, reports, innovative teaching concepts, specific offers as well as concrete initiatives already implemented.

The topics include approaches for the curricular integration of diversity aspects in German speaking countries, but also in the Netherlands and Australia, a description of a workshop on the integration of diversity in university didactics and further education of faculty members in Canada, students' views on the curricular integration of diversity, implementation of the Maternity Protection Act in anatomy courses, sex/gender differences in the assessment of interprofessional teaching formats, sex/gender differences in the perception of problems and challenges in the study environment, an evaluation of CIRS data with regard to the challenges that intercultural teams in care contexts are facing, and the promotion of gender awareness among male, white students in America.

The majority of the contributions is from German speaking countries, but we are pleased to also present international contributions from America, Australia, Canada and the Netherlands.

We hope that this special edition booklet will contribute to the current discussion and to the identification of existing research gaps and obstacles as well as to the joint development of approaches and solutions to increase diversity awareness and equality of opportunity in medical care.

## Acknowledgements

The editors would like to thank the German Association for Medical Association for making this special edition of the GMS Journal for Medical Education possible. A special thank goes to Beate Hespelein for her great commitment in managing the submitted articles and in coordinating the peer review process. We would also like to thank the authors and reviewers for their excellent cooperation and their great commitment. Without their work and contributions this special edition would not have been possible.

## Competing interests

The authors declare that they have no competing interests. 
